# Distinct cerebellar foliation anomalies in a *CHD7* haploinsufficient mouse model of CHARGE syndrome

**DOI:** 10.1002/ajmg.c.31595

**Published:** 2017-11-23

**Authors:** Danielle E. Whittaker, Sahrunizam Kasah, Alex P. A. Donovan, Jacob Ellegood, Kimberley L. H. Riegman, Holger A. Volk, Imelda McGonnell, Jason P. Lerch, M. Albert Basson

**Affiliations:** ^1^ Centre for Craniofacial and Regenerative Biology King's College London London United Kingdom; ^2^ Department of Clinical Science and Services Royal Veterinary College London United Kingdom; ^3^ Mouse Imaging Centre Hospital for Sick Children Toronto Ontario Canada; ^4^ Department of Comparative Biomedical Sciences Royal Veterinary College London United Kingdom; ^5^ Department of Medical Biophysics University of Toronto Toronto Ontario Canada; ^6^ MRC Centre for Neurodevelopmental Disorders King's College London London United Kingdom

**Keywords:** cerebellum, CHARGE syndrome, CHD7, fissures, foliation, mouse

## Abstract

Mutations in the gene encoding the ATP dependent chromatin‐remodeling factor, *CHD7* are the major cause of CHARGE (Coloboma, Heart defects, Atresia of the choanae, Retarded growth and development, Genital‐urinary anomalies, and Ear defects) syndrome. Neurodevelopmental defects and a range of neurological signs have been identified in individuals with CHARGE syndrome, including developmental delay, lack of coordination, intellectual disability, and autistic traits. We previously identified cerebellar vermis hypoplasia and abnormal cerebellar foliation in individuals with CHARGE syndrome. Here, we report mild cerebellar hypoplasia and distinct cerebellar foliation anomalies in a *Chd7* haploinsufficient mouse model. We describe specific alterations in the precise spatio‐temporal sequence of fissure formation during perinatal cerebellar development responsible for these foliation anomalies. The altered cerebellar foliation pattern in *Chd7* haploinsufficient mice show some similarities to those reported in mice with altered *Engrailed, Fgf8* or *Zic1* gene expression and we propose that mutations or polymorphisms in these genes may modify the cerebellar phenotype in CHARGE syndrome. Our findings in a mouse model of CHARGE syndrome indicate that a careful analysis of cerebellar foliation may be warranted in patients with CHARGE syndrome, particularly in patients with cerebellar hypoplasia and developmental delay.

## 1 INTRODUCTION

CHARGE syndrome is an autosomal dominant condition with an estimated prevalence of 1 in 10,000. CHARGE syndrome is characterized by a non‐random clustering of a complex array of congenital malformations (Jongmans et al., 2006; Lalani et al., 2006; Pagon, Zonana, & Yong, 1981). The discovery of loss‐of‐function mutations in the *CHD7* gene in patients with CHARGE syndrome (Janssen et al., 2012; Vissers et al., 2004), has led to significant progress in elucidating the developmental and molecular genetic mechanisms underlying specific phenotypes associated with CHARGE syndrome (Layman, Hurd, & Martin, 2010). However, CHARGE syndrome is characterized by significant variability in incidence and severity of specific abnormalities, which does not correlate with the nature of *CHD7* mutation (Basson & van Ravenswaaij‐Arts, 2015; Bergman, Janssen et al., 2011; Jongmans et al., 2008). These observations implicate other genetic or non‐genetic factors, or even stochastic effects as modifiers of disease severity. As CHD7 co‐factors and target genes are highly context‐dependent, the identity of these disease modifiers are likely to be distinct for each of the different phenotypes associated with CHARGE syndrome (Basson & van Ravenswaaij‐Arts, 2015).

Chromatin immunoprecipitation and subsequent sequencing (CHIP‐seq) studies demonstrate that CHD7 is recruited preferentially to distal gene regulatory elements, or enhancers, implying a role for CHD7 in enhancer‐regulated gene transcription (Engelen et al., 2011; Schnetz et al., 2009; Zentner et al., 2010). In vitro studies have provided direct experimental evidence for the ATP‐dependent nucleosome remodeling activity of CHD7. The introduction of CHARGE syndrome‐associated mutations in the ATP‐dependent chromatin remodeling and chromodomains of CHD7, tested in nucleosome remodeling assays, provided proof that chromatin remodeling activity is central to the pathogenesis of CHARGE syndrome (Bouazoune & Kingston, 2012). We recently reported changes in DNA accessibility at thousands of putative gene regulatory elements in *Chd7*‐deficient cerebellar neuron progenitors, providing in vivo evidence that altered nucleosome remodeling underlies specific phenotypes associated with CHD7 deficiency (Whittaker et al., 2017).

Patients with CHARGE syndrome frequently exhibit clinical signs pertaining to neurological dysfunction, which include developmental delay, motor incoordination, intellectual disability, and autistic features (Bergman, Janssen et al., 2011; Vesseur et al., 2016). Neuroanatomical anomalies have been reported in CHARGE syndrome (Becker, Stiemer, Neumann, & Entezami, 2001; Bergman, Bocca, Hoefsloot, Meiners, & van Ravenswaaij‐Arts, 2011; Issekutz, Prasad, Smith, & Blake, 2005; Lin et al., 1990; Sanlaville et al., 2006; Sanlaville & Verloes, 2007; Tellier et al., 1998). Lin et al. (1990) identified CNS anomalies in 55% (*n* = 26/47) and Tellier et al. (1998) in 79% (*n* = 37/47) of CHARGE syndrome patients. Sanlaville et al. (2006) reported CNS anomalies in 100% (*n* = 10/10) and Legendre et al. (2012) in 70% (*n* = 29/40) of pre‐term fetuses with *CHD7* mutations. Specific anomalies included hypoplasia of the olfactory bulbs and mild cerebellar vermis hypoplasia (Becker et al., 2001; Legendre et al., 2012; Sanlaville et al., 2006; Sanlaville & Verloes, 2007). Structural MRI analysis of a small cohort of CHARGE syndrome patients with *CHD7* mutations identified cerebellar vermis hypoplasia in 35% of patients, with evidence for abnormal foliation in 25% of patients (Yu et al., 2013).

Our studies on mouse mutants with either constitutive (gene‐trap) or conditional loss‐of‐function *Chd7* mutations have identified two temporally distinct roles for CHD7 during cerebellar development. During early to mid‐gestation, CHD7 is essential for the maintenance of high levels of *Fgf8* expression in the mid‐hindbrain organizer (Yu et al., 2013). Diminished FGF signaling contributes specifically to hypoplasia of the cerebellar vermis (Yu et al., 2013). During the peri‐ and early postnatal stages of development, *Chd7* is highly expressed in granule cell progenitors (GCps) on the surface of the cerebellar anlage, where it regulates cerebellar growth by controlling the proliferation, differentiation, and survival of this cell population (Whittaker et al., 2017).

Growth and foliation of the cerebellum are closely linked processes during perinatal development. Both are driven by GCp proliferation and therefore, disruption in GCp expansion usually affects both cerebellar size and the degree of foliation. Mutant mouse models with reduced cerebellar GCp proliferation, such as those with altered SHH signaling, exhibit cerebellar hypoplasia with a simplified pattern of foliation. Importantly, despite a marked reduction in cerebellar size, cardinal lobes, and therefore the presence and position of basic folia are retained, meaning that reduced cerebellar growth alone is insufficient to cause an alteration in the precise temporal and spatial sequence of fissure formation (Corrales, Blaess, Mahoney, & Joyner, 2006). The process of foliation is initiated by the formation of specific multicellular anchor points at the position of prospective fissures, identified by indentations on the surface of the developing cerebellum (Sudarov & Joyner, 2007). These fissures serve to divide the cerebellum into specific lobes across its anterior–posterior extent. Specific fissures appear in a stereotypical pattern at specific developmental time points in a highly regulated manner (Sudarov & Joyner, 2007). At completion of this process, the mammalian cerebellum is divided into ten cardinal lobules in the vermis (I–X) and four in the hemispheres (Simplex, CrusI, CrusII, Paramedian) (Larsell, 1952).

Although the exact molecular mechanisms that determine the position and timing of fissure formation have not been elucidated, it is clear that these features are under genetic control. The cerebellum is partitioned into zones, each with its own distinct gene expression patterns that likely pre‐figure and represent important functional subdivisions (Sillitoe & Joyner, 2007). The curious cerebellar phenotypes of spontaneous mouse mutants like meander tail (*mea/mea*) were the first to suggest that cerebellar regionalization was genetically determined. In this mouse, cellular disorganization is restricted to the anterior vermis up to the junction between lobules VI and VII (Norman, Fletcher, & Heintz, 1991; Ross, Fletcher, Mason, Hatten, & Heintz, 1990). Since then, specific genes that control foliation have been identified. For example, mutation of the Engrailed (En) genes, which encode homeobox transcription factors, alters the sequence, and position of fissure formation leading to changes in the shape and location of intervening folia (Cheng et al., 2010; Sudarov & Joyner, 2007).

Our previous analysis of cerebellar structure in *Chd7* heterozygous mice did not detect overt cerebellar vermis hypoplasia (Yu et al., 2013). However, only a small number of animals were examined in that study. Here, we report an analysis of a larger group of animals, which revealed mild, but significant, cerebellar hypoplasia and altered cerebellar foliation in approximately 65% (*n* = 12/18) of *Chd7* heterozygous mice.

## 2 MATERIALS AND METHODS

### 2.1 Mice

The *Chd7^xk403/+^* gene trap (*Chd7^gt/+^*) mouse line has been described (Randall et al., 2009) and was maintained on a B6D2F1 background. Genotyping was performed from ear or tail DNA. Mice were bred in the Biological Services Unit, King's College, London. The institutional Local Ethical Review Panel and the UK Home Office approved all experimental procedures (PPL70/6694 and PPL70/7506).

### 2.2 Histology

All samples were dissected in ice‐cold PBS, fixed overnight in 4% paraformaldehyde (PFA) at 4°C, dehydrated and embedded in paraffin wax. Serial, sagittal sections were cut (10 µm) and left to dry overnight at 42°C.

### 2.3 In situ hybridization

In situ hybridization was performed using previously described methods (Yaguchi et al., 2009). Digoxigenin‐labeled antisense probes *Fgf3* and *Fgf5* were generated using previously described constructs (Yaguchi et al., 2009).

### 2.4 Structural MRI

Adult mice (∼P60) were terminally anesthetized and intracardially perfused with 30 ml of 0.1 M PBS containing 10 U/ml heparin and 2 mM ProHance (Bracco Diagnostics Inc., Montreal, Quebec, Canada), a Gadolinium contrast agent, followed by 30 ml of 4% paraformaldehyde (PFA) containing 2 mM ProHance (Cahill et al., 2012; Spring, Lerch, & Henkelman, 2007). Perfusions were performed at a rate of approximately 60 ml/hr. After perfusion, the brain and remaining skull structures were incubated in 4% PFA + 2 mM ProHance overnight at 4°C and transferred to 0.1 M PBS containing 2 mM ProHance and 0.02% sodium azide for at least 1 month prior to MRI scanning (de Guzman, Wong, Gleave, & Nieman, 2016). A multi‐channel 7.0 Tesla MRI scanner (Agilent Inc., Palo Alto, CA) was used to image the brains within skulls. Sixteen custom‐built solenoid coils were used to image the brains in parallel (Bock et al., 2005; Lerch, Sled, & Henkelman, 2011). Parameters used in the anatomical MRI scans: T2‐weighted 3D fast spin‐echo sequence, with a cylindrical acquisition of k‐space, and with a TR of 350 ms, and TEs of 12 ms per echo for 6 echoes, 2 averages, field‐of‐view of 20 × 20 × 25 mm^3^ and matrix size = 504 × 504 × 630 giving an image with 0.040 mm isotropic voxels (Spencer Noakes, Henkelman, & Nieman, 2017). The current scan time required for this sequence is ∼14 hr. To visualize and compare any changes in the mouse brains the images were linearly (6 parameter followed by a 12 parameter) and non‐linearly registered together, and then iteratively linearly and non‐linearly aligned to each other to create a population atlas representing the average anatomy of the entire study sample. At completion of this registration, all scans had been deformed into alignment with each other in an unbiased fashion. As with typical deformation based morphometry, this allows for analysis of the deformations required to register the anatomy of each individual mouse into the final atlas space (Lerch et al., 2008; Nieman, Flenniken, Adamson, Henkelman, & Sled, 2006). The Jacobian determinants, as calculated through this analysis process, were used as measures of volume at each voxel and compared across genotypes.

### 2.5 Statistics

For MRI data, volumetric changes were calculated on a regional and a voxel‐wise basis. Regional volumes were determined using a pre‐existing classified MRI atlas encompassing 159 different structures throughout the brain (Dorr, Lerch, Spring, Kabani, & Henkelman, 2008; Steadman et al., 2014; Ullmann, Watson, Janke, Kurniawan, & Reutens, 2013). Statistical analyses were applied comparing the absolute and relative volume of these 159 different regions and on a voxel‐wise basis in the brains of control and *Chd7^gt/+^* mice. Multiple comparisons were controlled for using the False Discovery Rate (Genovese, Lazar, & Nichols, 2002).

## 3 RESULTS

### 3.1 Mild cerebellar hypoplasia and distinct foliation anomalies in *Chd7^gt/+^* mice

**Figure 1:**
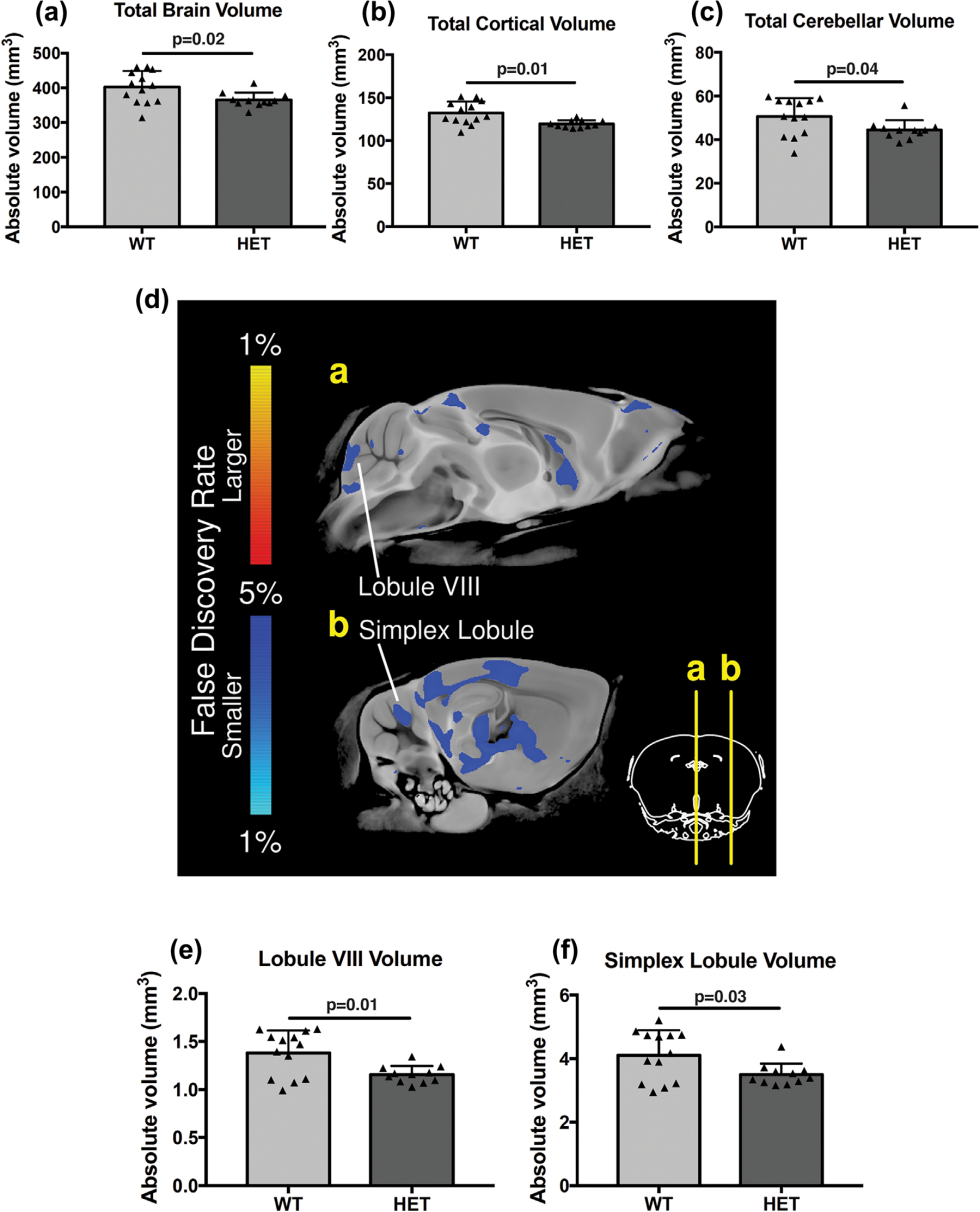
Brain, cortex, and cerebellar hypoplasia in *Chd7* haploinsufficient mice. (a) Absolute brain volumes (mm^3^) of *Chd7^+/+^* (WT, *n* = 13) and *Chd7^gt/+^* (HET, *n* = 11) adult mouse brains determined by high‐resolution structural MRI. (b) Total cortical volumes (mm^3^) of WT and HET adult mouse brains determined by high‐resolution structural MRI. (c) Total cerebellar volumes (mm^3^) of WT and HET adult mouse brains determined by high‐resolution structural MRI. (d) MRI images in the sagittal plane showing mid‐sagittal (a) and lateral (b) views of the brain, anterior to the right, with brain regions with significant (FDR < 0.05) volumetric differences between HET and WT mice colored according to the color scale. Note the hypoplastic regions in the posterior cerebellar vermis (lobule VIII) and anterior cerebellar hemispheres (Simplex lobule). (e) Lobule VIII volumes (mm^3^) of WT and HET adult mouse brains determined by high‐resolution structural MRI. (f) Simplex lobule volumes (mm^3^) of WT and HET adult mouse brains determined by high‐resolution structural MRI

Cerebellar hypoplasia has been reported in pre‐term CHARGE fetuses (*n* = 11/39, 28%) (Becker et al., 2001; Legendre et al., 2012; Sanlaville et al., 2006) and in a cohort of patients with *CHD7* mutations (*n* = 7/20, 35%) (Yu et al., 2013). As a first step toward identifying structural brain abnormalities in *Chd7* haploinsufficient mice in an unbiased manner, brains from 11 adult *Chd7^gt/+^* (HET) and 13 *Chd7^+/+^* wildtype (WT) littermate control mice were imaged by high resolution structural MRI. Volumetric analyses revealed a 9% reduction in mean total brain volume in *Chd7^gt/+^* mice compared to controls (Figure 1a). Mean cortical volume was likewise reduced by 9.7% (Figure 1b), in keeping with the general reduction in brain size. Mean cerebellar volume was reduced by approximately 12% in *Chd7^gt/+^* mice, compared to *Chd7^+/+^* controls (Figure 1c). Voxel‐wise, volumetric comparisons (Figure 1d) revealed the most significant (FDR < 0.05) changes in the posterior vermis (16% reduction in lobule VIII) and anterior hemisphere (15% reduction in simplex lobule) of the cerebellum (Figures 1e and 1f).

**Figure 2:**
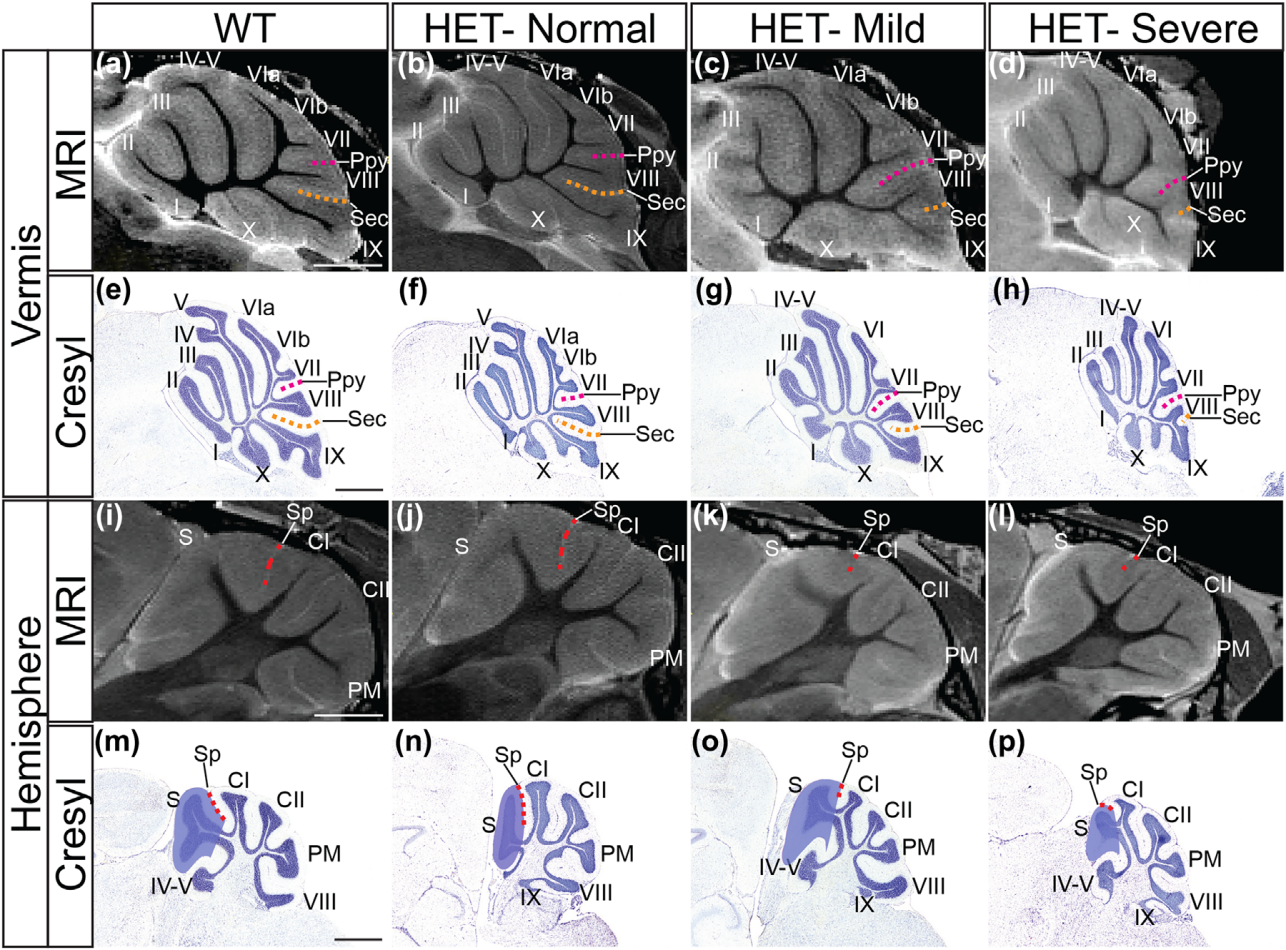
Subtle cerebellar foliation anomalies in *Chd7* haploinsufficient mice. (a–d) Representative mid‐sagittal MRI images of cerebella from *Chd7^+/+^* (WT) and *Chd7^gt/+^* (HET) mice, anterior to the left. Cerebellar lobules are labeled with Roman numerals according to Inouye and Oda (1980). Images from HET cerebella with normal foliation, subtle posterior shift of lobule VIII accompanied by deeper Ppy (broken purple line) and shallower Sec (broken orange line) fissures (mild), and most pronounced foliation phenotype characterized by small and posteriorly shifted lobule VIII associated with shallow Sec fissure (severe) are shown. (e–h) Representative Cresyl violet‐stained sagittal sections demonstrating the foliation patterns in (a–d) at higher resolution. (i–l) Representative sagittal MRI images of lateral cerebella from *Chd7^+/+^* (WT) and *Chd7^gt/+^* (HET) mice. Cerebellar lobules are labeled with Roman numerals as above. Images from HET cerebella with normal foliation (HET‐normal), and shallower Sp (broken red line) fissures (HET‐mild and HET‐severe) are shown. (m–p) Representative Cresyl violet‐stained sagittal sections demonstrating the foliation patterns in (i–l) at higher resolution. Purple shading outlines the simplex lobule (m–p). Ppy, prepyramidal; Sec, secondary; Sp, superior posterior; S, simplex; CI, CrusI; CII, CrusII; PM, paramedian. Scale bar = 1 mm

To examine whether these volumetric changes in specific cerebellar areas were related to specific changes in cerebellar foliation, cerebella were examined on sagittal MRI slices. The assessment of images from the cerebellar vermis (Figure 2a–d) identified a subtle foliation anomaly in 64% (*n* = 7/11) of *Chd7^gt/+^* mice (Figures 2c and 2d), with the remaining 36% (*n* = 4/11) of mutants (Figure 2b) exhibiting a normal foliation structure. The severity of the observed phenotype appeared to differ between individual mice. Mildly affected mice (*n* = 3, Figure 2c) had a deeper prepyramidal (Ppy) fissure and correspondingly shallower secondary (Sec) fissure, such that lobule VIII was located in a more posterior position. This phenotype was even more striking in the severely affected group (*n* = 4, Figure 2d), in which a marked reduction in the size of lobule VIII was accompanied by a more pronounced posterior shift of this lobule. Cerebellar hypoplasia also appeared to be more pronounced in the severely affected group, an observation that was supported by volumetric analysis, which showed a reduction in mean cerebellar volume of 17% in this group compared to wild type (Table 1).

**Table 1:** Cerebellar volumes (mm^3^) from high resolution MRI

Region	WT, *n* = 13	All HETs, *n* = 11	HET‐normal, *n* = 4	HET‐mild, *n* = 3	HET‐severe, *n* = 4
Total cerebellum	50.6415 ± 2.35	44.5081 ± 1.33, −12%*	46.8324 ± 3.03, −8%	44.7637 ± 0.49, −12%	41.9921 ± 1.76, −17%
Lobule VIII	1.3815 ± 0.06	1.1561 ± 0.03, −16%	1.1865 ± 0.06, −14%	1.1520 ± 0.04, −17%	1.1288 ± 0.05, −18%
Simplex lobule	4.1082 ± 0.22	3.4958 ± 0.1, −15%	3.6962 ± 0.23, −10%	3.4499 ± 0.09, −16%	3.3298 ± 0.13, −19%

Mean cerebellar volumes ± SEM for each group, as determined by MRI.

* % difference from wildtype volumes are shown for each group.

To visualize the foliation abnormalities at higher resolution, a careful histological analysis was performed on sections from *Chd7^gt/+^* and *Chd7^+/+^* cerebella at P21 (Figure 2e–h). In wild‐type mice, lobule VIII always appears as a distinct lobule, separated by the Ppy and Sec fissures from the adjacent VIth and IXth lobules, respectively (Figure 2e). In agreement with the MRI analysis, a proportion (*n* = 2/7) of P21 *Chd7^gt/+^* cerebella exhibited a normal foliation pattern (Figure 2f). The remaining *Chd7^gt/+^* mice presented with either a small posterior shift of lobule VIII along lobule IX (*n* = 3/7, Figure 2g), or a more pronounced shift (*n* = 2/7, Figure 2h), accompanied by clear hypoplasia of this lobule. Combining the MRI and histology data, the total incidence of the foliation change affecting lobule VIII in the vermis is 67% (*n* = 12/18).

A similar analysis of MRI images and histological sections through the lateral cerebellum also revealed a foliation defect in the hemispheres (Figure 2i–p). Specifically, the superior posterior (Sp) fissure that separates the simplex lobule from Crus I (CI), appeared to be shallower in some mutants, leading to partial “fusion” of these lobules in the anterior cerebellar hemispheres (Figures 2k, 2l, 2o, and 2p). This hemisphere‐specific foliation defect was identified in the same mice exhibiting foliation defects in the vermis and the overall penetrance of this foliation phenotype in the hemispheres is therefore also 67% (*n* = 12/18).

**Figure 3:**
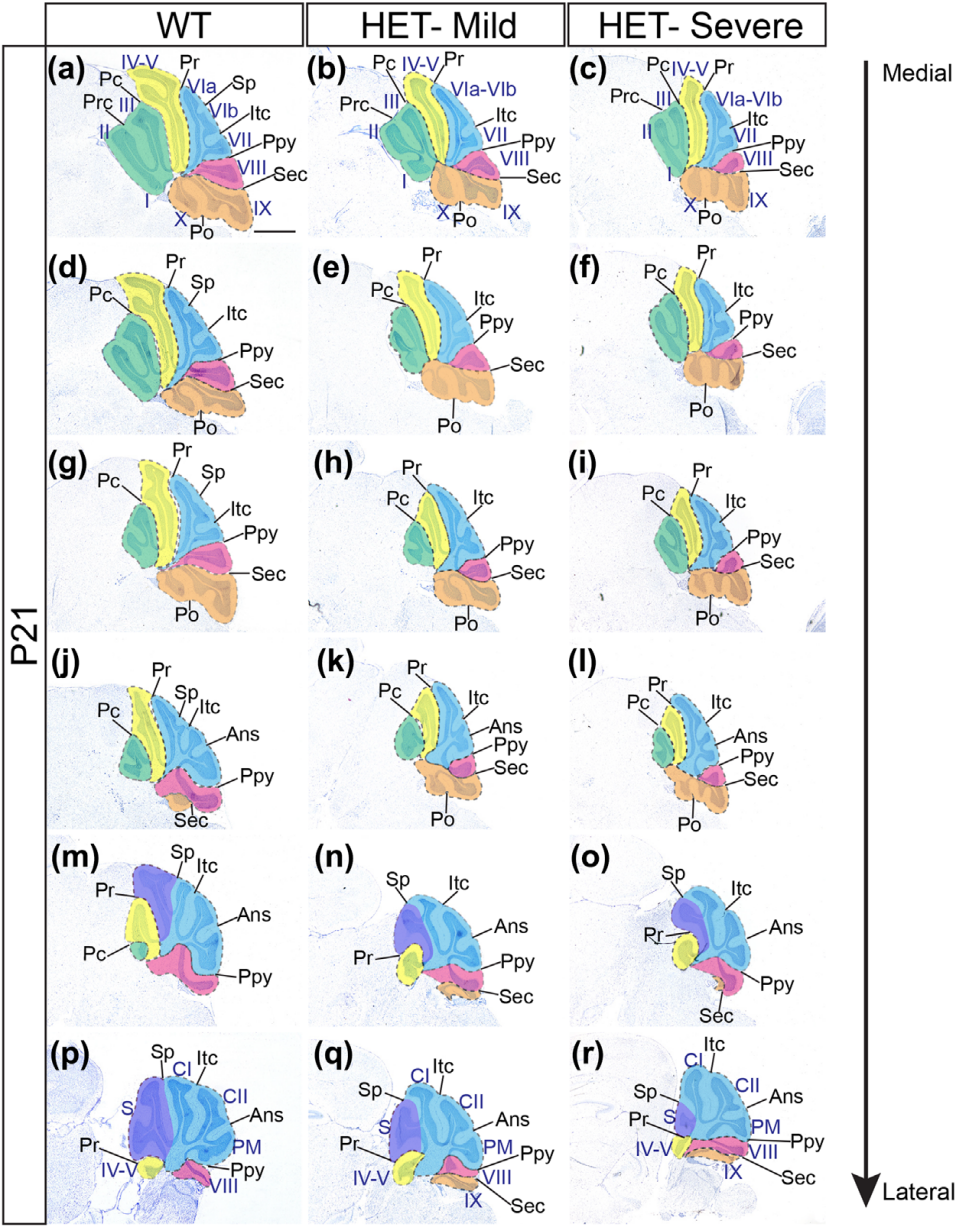
Medio‐lateral sections through representative wildtype and *Chd7* heterozygous cerebella from P21 mice to visualize foliation anomalies. (a–r) Cresyl violet‐stained sagittal sections through the cerebellum of *Chd7^+/+^* (WT) and *Chd7^gt/+^* (HET‐mild, HET‐severe) mice, anterior to the left. Medial sections are at the top, with more lateral sections below. Representative images from mildly affected heterozygous (b, e, h, k, n, q) and severely affected (c, f, i, l, o, r) heterozygous mice animals are shown. Folia in the vermis are labeled with Roman numerals as in Figure 2. Green shading indicates lobules I–III, yellow indicates IV–V, blue indicates VI–VII, pink indicates VIII and orange IX and X. Note the posterior shift of lobule VIII in the cerebellar vermis of *Chd7^gt/+^* mice (b, c, e, f, h, i) and partial fusion of the simplex lobule with CI in the hemisphere (q and r) highlighted by purple shading, accompanied by shallower Sp fissure. Pc, preculminate; Pr, primary; Ppy, prepyramidal; Sec, secondary; Po, posterolateral; Sp, superior posterior; Itc, intercrural; Ans, ansoparamedian. Scale bar = 1 mm

To visualize these foliation anomalies across the whole cerebellum, representative histological sections along the medial‐lateral extent of *Chd7^gt/+^* and *Chd7^+/+^* cerebella were compared (Figure 3). The alterations in Ppy and Sec fissures, with associated hypoplasia of lobule VIII (false colored in pink in Figure 3) can clearly be seen in mid‐sagittal sections from both *Chd7^gt/+^* mutants (Figures 3b and 3c). This phenotype was present along the entire vermis (Figure 3d–i) and paravermis (Figure 3j–l). The shallower Sp fissure and hypoplastic simplex lobule “fused” to CI can be discerned in the hemispheres from both groups (Figure 3m–r).

Together, these analyses showed that *Chd7* haploinsufficiency in mice is associated with mild cerebellar hypoplasia, and identified incompletely penetrant roles for CHD7 in regulating cerebellar foliation in the vermis and hemispheres. The specific foliation anomalies observed in these mutants appeared to be responsible for the hypoplasia of the VIIIth and simplex lobules.

### 3.2 CHD7 coordinates the precise temporal sequence of fissure formation during perinatal development

The foliation defects identified in *Chd7^gt/+^* mice were suggestive of a role for CHD7 in regulating the timing and/or positioning of individual cerebellar fissures. A disruption in the specific sequence of fissure formation during development can lead to predictable alterations in the location, size and shape of the intervening folia (Cheng et al., 2010; Orvis et al., 2012; Sudarov & Joyner, 2007). We therefore hypothesized that changes in the timing of formation and/or position of the prepyramidal and secondary fissures in the posterior vermis may be responsible for the posterior shift of lobule VIII in the *Chd7* mutants. To determine the precise sequence and timing of fissure formation in *Chd7^gt/+^* mice, cerebella were analyzed at key developmental time points.

**Figure 4:**
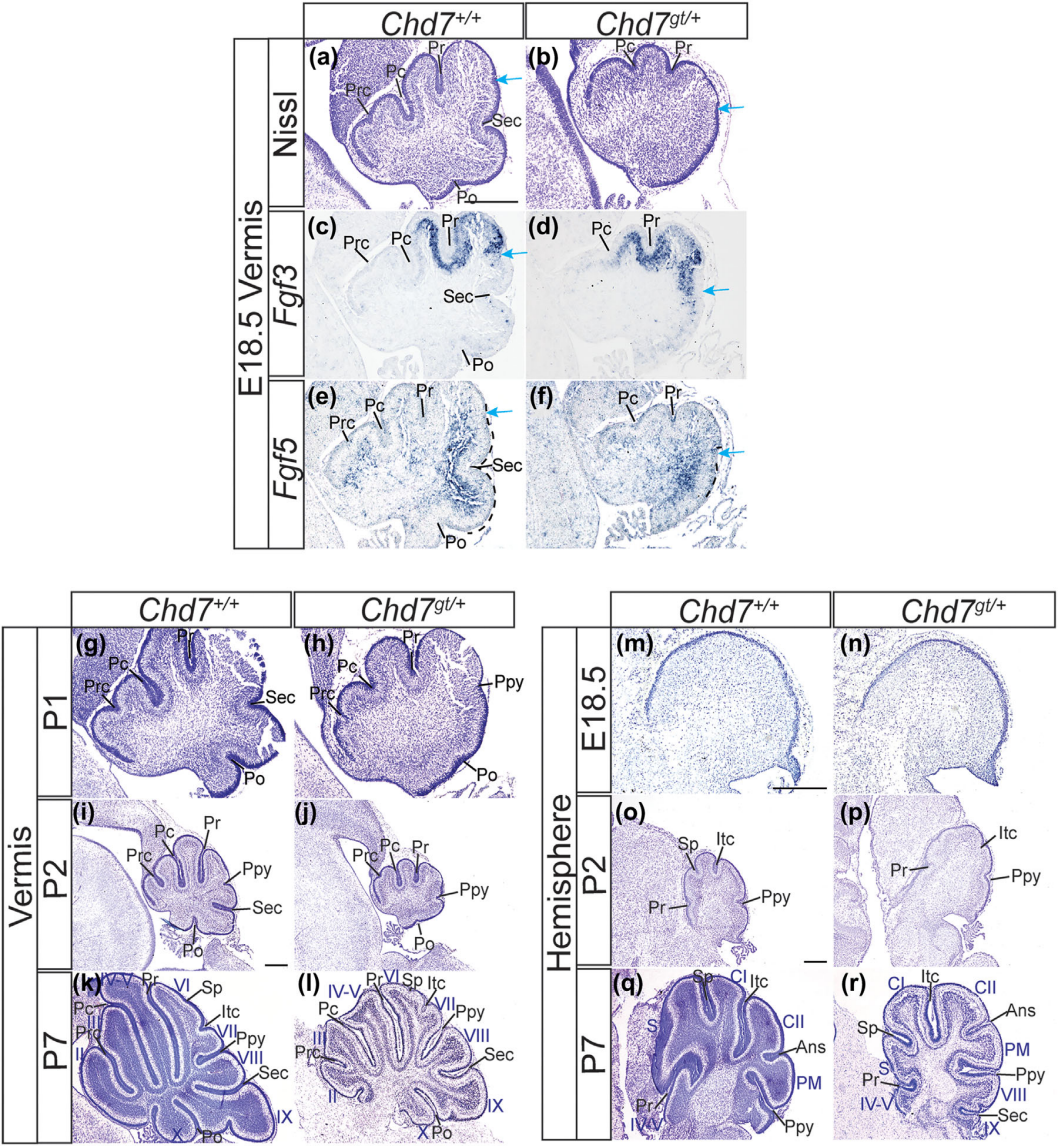
CHD7 regulates the timing and sequence of fissure formation during cerebellar development. (a and b) Cresyl violet stained sagittal sections of the cerebellum of *Chd7^+/+^* and *Chd7^gt/+^* mice at E18.5, anterior to the left. Developing fissures are labeled. The presumptive Ppy fissure is indicated by a blue arrow. (c and d) In situ hybridization to visualize *Fgf3* transcripts in E18.5 cerebellar sections as above. Note the posterior limit of *Fgf3* expression at the presumptive site of Ppy fissure formation (blue arrow). (e and f) *Fgf5* expression detected by in situ hybridization on E18.5 cerebellar sections as above. Note the area of high *Fgf5* expression in the posterior cerebellum across the Sec fissure (e) and the anterior limit of this *Fgf5* expression domain at the presumptive site of Ppy fissure formation (blue arrow). (g–l) Cresyl violet stained sagittal sections of the cerebellar vermis of *Chd7^+/+^* and *Chd7^gt/+^* mice at the indicated postnatal stages, anterior to the left. Developing fissures are labeled. (m–r) Cresyl violet stained sagittal sections of the cerebellar hemispheres of *Chd7^+/+^* and *Chd7^gt/+^* mice at the indicated postnatal stages, anterior to the left. Developing fissures are labeled. Prc, precentral; Pc, preculminate; Pr, primary; Ppy, prepyramidal; Sec, secondary; Po, posterolateral; Sp, superior posterior; Itc, intercrural. Scale bar = 1 mm

In control mice, the fissures developed in a highly coordinated and reproducible temporal series as previously described (Sudarov & Joyner, 2007). At E18.5, the three cardinal fissures that are initiated a day earlier, the preculminate (Pc), primary (Pr), and secondary (Sec), were clearly visible (Figure 4a). At E18.5, an additional fissure, the posterolateral (Po) fissure, begins to form (Figure 4a). Together, these four cardinal fissures partition the cerebellum into five cardinal lobes (Figure 4a). Comparing sections from *Chd7^gt/+^* with these controls at E18.5 revealed a general delay in fissure formation in the vermis of *Chd7^gt/+^* mice, with shallower pc and pr fissures and absent sec and po fissures (Figure 4b). An indentation in the posterior vermis of *Chd7^gt/+^* cerebella indicated the initiation of a new fissure (blue arrow, Figure 4b). The position of this presumptive fissure relative to others suggested that this indentation represented the first signs of formation of either the prepyramidal or secondary fissures. To determine the identity of this fissure, we used molecular markers with restricted expression domains in the perinatal cerebellum. We have previously shown that *Fgf3* and *Fgf5* expression patterns are mutually exclusive at this stage (Yaguchi et al., 2009), with the border between *Fgf3* expression anteriorly and *Fgf5* expression posteriorly corresponding to the position of Ppy fissure formation (blue arrow, Figures 4a, 4c, and 4e). Examination of *Fgf3* and *Fgf5* expression in the E18.5 *Chd7^gt/+^* vermis, found that *Fgf3* expression was shifted posteriorly at the expense of *Fgf5* expression (Figures 4d and 4f). We therefore concluded that the fissure beginning to form in the mutant cerebella was the prepyramidal fissure that was being initiated at a more posterior position than normal (Figure 4b). The Sec fissure, which normally forms in the centre of the *Fgf5* expression domain in the posterior vermis (Figure 4e), was absent in the mutants (Figure 4f). These abnormalities in fissure formation was detected in 60% (*n* = 3/5) of the *Chd7^gt/+^* mice analyzed at E18.5 and P0, which represents a similar frequency to the foliation defects observed in adult heterozygous *Chd7* mutants.

By following fissure formation in histological sections at postnatal stages, we confirmed a pronounced delay in formation of the posterolateral and Sec fissures. The posterolateral fissure first became evident in affected *Chd7* haploinsufficient mice by P1 (Figure 4h), >2 days later than its formation in control mice at E18.5 (Figure 4a). By P2, when the secondary fissure is already prominent in controls (Figure 4i), these mutants still completely lacked any signs of this fissure (Figure 4j). The more posterior position of the Ppy fissure in mutants, compared to controls was also evident at P2 (Figures 4i and 4j). By P7, a Sec fissure was present in the mutants (Figure 4l), indicating that this fissure forms between P2 and P7, several days later than controls where this fissure starts forming at E17.5. Taken together, this analysis shows that formation of the preculminate, primary, secondary, and posterolateral fissures is delayed in some *Chd7* haploinsufficient mice, while the prepyramidal fissure is shifted to a more posterior position. We conclude that this posterior shift in the Ppy fissure, together with the striking delay in formation of the Sec fissure, are responsible for the abnormal position and hypoplasia of lobule VIII in the vermis.

Similar to findings in the vermis, we observed a general delay in fissure formation in the hemispheres from *Chd7^gt/+^* mice. Fissure formation in the hemisphere first becomes visible at postnatal stages in the mouse. The intercrural (Itc) and superior posterior (Sp) fissures form simultaneously at P1 while the hemisphere specific ansoparamedian (Ans) fissure forms at P5 (Cheng et al., 2010). A general delay in fissure formation was noted at P2 in *Chd7^gt/+^* mice, with fissures shallower and less developed than those in control mice (Figures 4o and 4p). In addition, the temporal sequence of fissure formation appeared disrupted in the hemispheres of *Chd7^gt/+^* mice. Whereas the Itc fissure was present in *Chd7^gt/+^* mice at P2 (Figure 4p), the Sp fissure was absent at this stage. Both fissures were present at P7 (Figure 4r), indicating that formation of the Sp fissure was specifically delayed. As this fissure separates the simplex and CI lobules, delayed formation results in a shallower Sp fissure at later stages, incomplete separation of the simplex and CI lobules, and hypoplasia of the simplex lobule. These abnormalities in the hemispheres were present in 60% of *Chd7^gt/+^* brains examined, in agreement with the incidence of foliation defects observed in adults.

Overall, these data demonstrate that foliation defects in *Chd7* haploinsufficient mice result from abnormalities in the timing and position of fissure formation during cerebellar development. The regulation of the precise temporal sequence of fissure formation in the perinatal cerebellum by CHD7 represents a previously unidentified role for this chromatin‐remodeling factor in cerebellar foliation.

## 4 DISCUSSION

Here, we report the first small‐scale, unbiased structural brain MRI study of a *Chd7* haploinsufficient mouse model. Several intriguing observations were made, which included a general reduction in brain size in these mutants that appears to affect several brain regions. These findings warrant further examination, including a careful longitudinal study of brain growth to determine to what extent brain anomalies are related to the general growth retardation typical of CHARGE syndrome. Quantitative structural MRI imaging in CHARGE syndrome patients will be important to identify specific brain regions most sensitive to *CHD7* haploinsufficiency during human brain development, as altered growth trajectories and volumetric changes in specific brain regions may underlie specific neuropsychiatric symptoms associated with CHARGE syndrome.

### 4.1 CHD7 controls cerebellar foliation independently from cerebellar growth

Previous studies have shown that cerebellar foliation occurs during perinatal development and is driven by extensive proliferation of cerebellar GCps (Sudarov & Joyner, 2007). The analysis of GCp‐specific *Chd7* conditional mutant mice has identified important roles for CHD7 in regulating GCp apoptosis, proliferation and differentiation in the perinatal cerebellum (Feng et al., 2017; Whittaker et al., 2017). However, a reduction in GCp expansion is by itself not sufficient to explain the cerebellar foliation defects described here. Although some fissures and folia show a general delay in their formation, which could be accounted for by reduced GCp proliferation, our data are consistent with a specific role for CHD7 in regulating the precise position and timing of fissure formation, which ultimately alters the position, size and shape of specific folia.

### 4.2 Specific genetic pathways controlling cerebellar foliation may be disrupted in CHARGE syndrome

The wide range of phenotypes associated with CHARGE syndrome is thought to primarily be the consequence of tissue‐specific dysregulation of gene transcription during development (Basson & van Ravenswaaij‐Arts, 2015; Engelen et al., 2011; Schnetz et al., 2009; Schnetz et al., 2010). It has been proposed that mutations or variants in genes that are regulated by or otherwise interact with CHD7 may contribute to, or modify, the phenotypic outcome of a mutation in *CHD7* (Basson, 2014). Mouse models have provided compelling evidence in support of this hypothesis, linking CHD7 to many important developmental pathways. For instance, *Fgf8* interacts with *Chd7* during early cerebellar vermis development (Yu et al., 2013), and *Chd7* interacts with *Tbx1* during arch vessel development (Randall et al., 2009). As cerebellar foliation is under genetic control (see section 1), it seems likely that genes controlling this process may be regulated by CHD7 and interact with *Chd7*.

The foliation defects identified in *Chd7^gt/+^* mice share some features with *En1/En2* deficient mice (Cheng et al., 2010; Joyner, Herrup, Auerbach, Davis, & Rossant, 1991; Millen, Wurst, Herrup, & Joyner, 1994; Orvis et al., 2012; Sgaier et al., 2007). The Engrailed genes encode homeodomain containing transcription factors that are master regulators of cerebellar patterning (Desplan, Theis, & O'Farrell, 1985; Joyner, Kornberg, Coleman, Cox, & Martin, 1985; Joyner & Martin, 1987; Sillitoe, Stephen, Lao, & Joyner, 2008; Sillitoe, Vogel, & Joyner, 2010). These genes are critical for normal cerebellar development, even after their early role in maintaining *Fgf8* expression in the isthmic organizer is complete. In fact, in later stages of cerebellar development, *En1/2* are essential for anterior‐posterior and medial‐lateral patterning of the cerebellum (Cheng et al., 2010; Joyner et al., 1991; Sgaier et al., 2007; Sillitoe et al., 2008; Sillitoe et al., 2010). Interestingly, our recent RNA‐seq analysis of *Chd7*‐deficient cerebellar GCps showed that *En1* expression was upregulated in these cells, implicating CHD7 as a potential repressor of *En1* expression (Whittaker et al., 2017). It is tempting to speculate that dysregulated *En1* expression contributes to the specific foliation anomalies we have identified in *Chd7*‐deficient mice. A role for CHD7 in regulating Engrailed gene expression would also be consistent with its conserved function as a Trithorax family member. Trithorax proteins regulate the expression of homeobox genes involved in regional identity and patterning during development (Schuettengruber, Martinez, Iovino, & Cavalli, 2011; Yu et al., 2013).

We previously showed that *Fgf8* expression in the isthmic organizer is reduced in *Chd7^gt/+^* embryos (Yu et al., 2013). We have also reported that *Fgf8* is expressed in the late embryonic (E16.5) and early postnatal cerebellum at the site of the developing secondary fissure (Yaguchi et al., 2009). Sato and Joyner have shown that *Fgf8* deletion from ∼E12 of development results in mice with a posteriorly located lobule VIII (Sato & Joyner, 2009), the identical foliation change we report here in *Chd7^gt/+^* mice. These findings suggest the intriguing possibility that a prolonged reduction in FGF8 signaling in *Chd7^gt/+^* embryos may also contribute to this specific foliation anomaly, in addition to its established role during early cerebellar development.

The cerebellar vermis anomalies identified in CHARGE syndrome patients share some phenotypic similarities with Dandy Walker malformation (DWM), the most frequent congenital cerebellar malformation in the human population (Barkovich, Millen, & Dobyns, 2009; Yu et al., 2013). Specifically, documented neuroanatomical malformations in CHARGE syndrome patients include cerebellar vermis hypoplasia with anti‐clockwise rotation away from the brainstem and a large posterior fossa (Yu et al., 2013). Other common features of CHARGE syndrome have also been identified in patients with DWM, including developmental delay and ataxia. Mutations encompassing *Zic1* and *Zic4* loci have been implicated in DWM and mouse models phenocopy many features of DWM (Blank et al., 2011). Interestingly, anterior hemisphere foliation defects in *Zic* gene‐deficient mice show similarities with the foliation defect noted in the anterior hemisphere of *Chd7^gt/+^* mice (Figures 2o and 2p). Specifically, *Zic1^−/−^* and *Zic1^+/−^*; *Zic2^+/−^* mice lack the superior posterior fissure in the anterior cerebellar hemisphere and Simplex and CrusI lobules are fused (Aruga, Inoue, Hoshino, & Mikoshiba, 2002). It will be of interest to quantify *Zic* gene expression in the anterior cerebellar hemispheres of *Chd7^gt/+^* mice to establish whether *Zic* expression in the developing anterior cerebellar hemisphere is sensitive to *Chd7* haploinsufficiency.

The phenotypic variability we report in *Chd7^gt/+^* mice suggests that this aspect of CHARGE syndrome can be modeled in a mouse model. The maintenance of these mice on an F1, rather than single inbred genetic background, may certainly contribute to higher levels of phenotypic variation (Keane et al., 2011). In addition, *Chd7^gt/+^* embryos may be more susceptible to unknown, stochastic effects on developmental gene expression.

### 4.3 Functional consequences of foliation anomalies in CHARGE syndrome

It is worth considering whether the relatively mild cerebellar anomalies contributes to specific neurological or psychiatric aspects of CHARGE syndrome. A couple of pertinent findings suggest the possibility that lobule VIII‐specific foliation defects may indeed contribute to some of the deficits in motor coordination and learning frequently associated with CHARGE syndrome (Admiraal & Huygen, 1997; Bergman, Janssen et al., 2011; Sanlaville & Verloes, 2007). Lobule VIII is active during sensorimotor tasks (Stoodley & Schmahmann, 2009; Stoodley, Valera, & Schmahmann, 2012) and abnormal motor learning and function have been reported in *En2* mutant mice, which have cerebellar vermis foliation defects similar to *Chd7^gt/+^* mice (Cheh et al., 2006; Gerlai, 1996; Joyner et al., 1991; Millen et al., 1994). However, to date, a correlation between motor dysfunction in CHARGE patients and cerebellar pathology has not been established. While cerebellar hypoplasia in CHARGE syndrome could influence gait or motor learning, there is a high prevalence (94% of CHARGE patients) of semicircular canal anomalies leading to vestibular dysfunction (Abadie et al., 2000). The vestibular apparatus is important in psychomotor development and therefore, it remains difficult to discern the precise contribution of cerebellar hypoplasia to the difficulties in postural and axial motor control in these patients. Previous studies imply that vestibular dysfunction alone is not responsible for gait abnormalities in patients and therefore further investigation of cerebellar contribution is necessary (Abadie et al., 2000; Wiener‐Vacher, Amanou, Denise, Narcy, & Manach, 1999).

The cerebellar hemispheres have been implicated in higher cognitive processes (Kelly & Strick, 2003; Stoodley & Schmahmann, 2009; Stoodley & Schmahmann, 2010; Stoodley et al., 2012) and thus, the foliation defects identified in the anterior cerebellum tentatively suggest a connection between these cerebellar defects and intellectual disability and autistic phenotypes associated with CHARGE syndrome. Separately, the relationship between cerebellar anomalies and autism is well established and in contrast to other brain regions, gross and microscopic changes in the cerebellum are most frequently associated with autism (Becker & Stoodley, 2013). However, we previously reported that cerebellar hypoplasia and foliation defects in *Chd7* GCp‐specific conditional mouse mutants, alone were not sufficient to lead to social deficits (Whittaker et al., 2017). The contribution of cerebellar dysfunction to autistic phenotypes in patients also remains unclear.

In conclusion, we have identified mild cerebellar hypoplasia and distinct cerebellar foliation anomalies in *Chd7^gt/+^* mice. Our findings imply a specific function for CHD7 in controlling the spatiotemporal initiation of cerebellar fissures and show that normal fissure formation requires bi‐allelic *Chd7* expression, consistent with the haploinsufficient nature of CHARGE syndrome. The incomplete penetrance and expressivity of these phenotypes are also consistent with the observed phenotypic spectrum of CHARGE syndrome. As with other phenotypes, the identification of possible genetic and environmental modifiers that interact with *Chd7* to regulate cerebellar foliation will be of significant interest.
